# Penetration of Silver Diamine Fluoride in Deep Carious Lesions of Human Permanent Teeth: An In Vitro Study

**DOI:** 10.1155/2021/3059129

**Published:** 2021-12-22

**Authors:** Jutharat Manuschai, Supitcha Talungchit, Supawadee Naorungroj

**Affiliations:** Department of Conservative Dentistry, Faculty of Dentistry, Prince of Songkla University, Hat Yai, Songkhla, Thailand

## Abstract

**Background:**

When silver diamine fluoride (SDF) is used in conjunction with conservative caries removal in deep carious lesions, the distribution depth of silver is critical for safety and effectiveness.

**Objective:**

The purpose of this study is to determine the effect of selected caries removal on silver penetration when 38% SDF is applied to deep carious lesions in permanent teeth.

**Methods:**

Extracted permanent teeth with caries extending to the inner third of the dentin were used (*N* = 18). The periphery of the carious lesion was completely removed to the dentinoenamel junction (DEJ). In group A (*n* = 9), no further removal of carious tissue was performed, leaving necrotic dentin inner to the DEJ, whereas in group B (*n* = 9) superficial necrotic dentin was completely removed until leathery, slightly moist, reasonably soft dentin remained. SDF was applied for 3 minutes in both groups. Microcomputer tomography (micro-CT) and field emission scanning electron microscopy coupled with energy-dispersive X-ray spectroscopy (FESEM-EDS) were used to measure mineral density and silver distribution. The silver penetration depth/lesion depth (PD/LD) ratio was calculated for each sample. The Mann–Whitney *U* test was used to compare differences between the two groups.

**Results:**

The micro-CT analysis showed that the PD/LD ratios of group B (1.07–2.29) were marginally greater than those of group A (1.00–1.31). However, a statistically significant difference was not observed (*p*value = 0.5078). When stratified by remaining dentin thickness (RDT), the PD/LD ratios of group B were still greater than those of group A only when RDT was >500 *µ*m. The FESEM-EDS analysis indicated that silver particles precipitated throughout the entire thickness of the carious lesions.

**Conclusion:**

Applying SDF on a deep carious lesion and leaving the necrotic dentin pulpally did not affect silver penetration. However, the extent to which silver penetrates the remaining dentin beneath the lesions is dependent on the amount and characteristics of that dentin.

## 1. Introduction

Maintaining pulp vitality is an important aspect for deep caries management. Evidence-based practice suggests stepwise excavation and selective caries removal to soft dentin to reduce the risk of mechanical pulp exposure [1, 2]. The intact dentin barrier not only protects the dental pulp but also releases growth factors to stimulate a reparative process [3]. Clinically, it is difficult to determine the precise amount of carious tissue overlying the pulp while there is a lack of consensus on how much caries can be left without any adverse consequences. Therefore, decision making regarding the need for reentry, which increases the risk of pulp exposure, is subjective. If the sealed active carious lesion could turn into an inactive lesion completely and pulp-dentin defenses occur effectively regardless of the amount of residual carious lesion, a one-step incomplete caries removal procedure in deep cavities may be more favorable.

When tertiary dentinogenesis is induced, the sealed active lesion must be harmless to the pulp. Previous studies have shown that placing biocompatible pulp capping materials with antimicrobial action and dentinogenesis induction properties such as calcium hydroxide along with a tightly sealed restoration reduces bacterial viability and promotes tertiary dentin formation, resulting in successful preservation of the pulp vitality [4, 5]. Despite serving as the gold standard for pulp capping, tunnel defects in reparative dentin, no adhesion to dentin, and high solubility are calcium hydroxide's major drawbacks [6]. The use of calcium hydroxide, therefore, is restricted to a thin layer in a specific area close to the pulp. As a result, the therapeutic effects of calcium hydroxide might not cover the entire area of the carious lesion.

Silver diamine fluoride (SDF) may be a promising agent to use adjunctively with the selective caries removal method in deep cavities. Current studies suggest that the action modes of SDF include (i) inhibiting cariogenic bacterial growth [7–12], (ii) slowing down the demineralization of carious dentin [7–9, 13], (iii) promoting the remineralization of demineralized dentin [7, 13–15], and (iv) inhibiting collagen degradation [8, 13]. In this regard, the extent of silver particle penetration and distribution is a crucial factor for safety and effectiveness, especially when applying SDF in deep carious lesions. A major concern with the use of SDF in deep carious lesions is that SDF components may irritate the pulp while leaving necrotic tissue that may compromise the cariostatic effects of SDF.

The evidence on this issue is limited and has been reported only in primary teeth [16]. The aim of this *in vitro* study was to assess the silver penetration into deep carious lesions in permanent teeth treated with 38% SDF, comparing two methods of selective caries removal. The null hypothesis was that leaving necrotic dentin did not affect the silver penetration into carious lesions.

## 2. Materials and Methods

### 2.1. Sample Collection

The protocol was approved by the Ethics Committee of the Faculty of Dentistry, Prince of Songkla University (EC6302-006). Extracted permanent teeth with deep cavities were rinsed thoroughly with tap water, kept in a plastic container, and then stored frozen in a refrigerator at –20°C within 5 hours of being extracted [17]. The carious lesion depth was assessed with a periapical radiograph. The inclusion criteria were permanent teeth with carious lesions extending to the inner third of the dentin. A total of 18 samples were included (Supplementary Table 1).

At the beginning of each experiment, the sample was placed in a single plastic container containing a wet paper towel placed in a lower partition to keep the storage condition under 100% humidity at room temperature (24°C) for 14 hours.

### 2.2. Caries Removal Protocol

**Figure 1:**
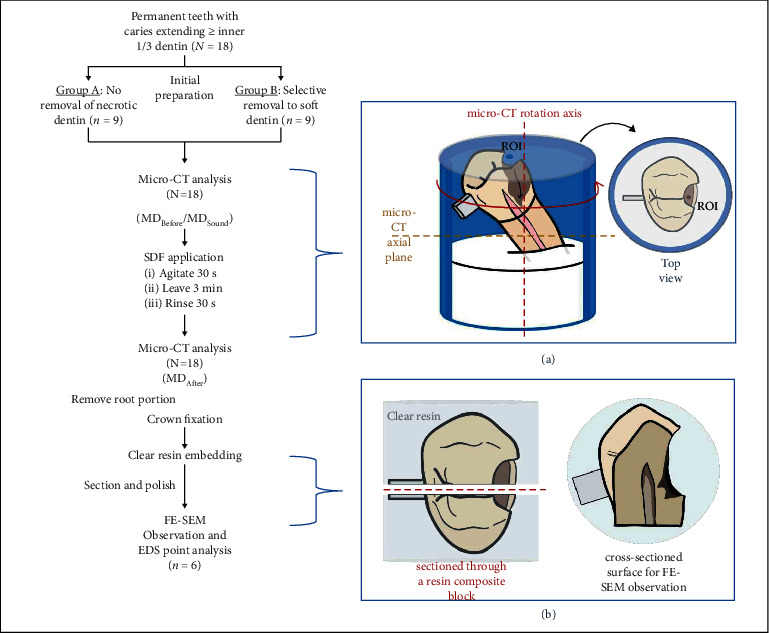
Illustration of the experimental design. (a) The position of the sample for micro-CT scanning; ROI (region of interest). (b) The direction of the sample cross section.

Figure 1 shows the design of this experimental study. Eighteen samples were divided into two groups using the minimization technique. Carious tissue on enamel was completely removed using round diamond burs operated at high speed under water cooling. Next, peripheral carious dentin at the dentinoenamel junction (DEJ) was completely removed in both groups using stainless steel round burs (size 010–014), resulting in a sound dentin zone of at least 1 mm from the DEJ. In group A, no further removal of carious tissue was carried out, leaving necrotic dentin which was very soft and wet inside the DEJ (*n* = 9). In group B, superficial necrotic dentin was completely removed using spoon excavators, reaching leathery, slightly moist, and reasonably soft dentin [18] (*n* = 9).

### 2.3. SDF Application Protocol

Each lesion was applied using a microbrush saturated with 12–14 *µ*l of 38% SDF solution (Topamine, 25% w/v silver ion; Dentalife, Victoria, AUS, lot No. B0321) with agitation for 30 seconds and left for 3 minutes. Then, each lesion was rinsed with water from a triple syringe for 30 seconds and gently dried with an air syringe for 10 seconds.

### 2.4. Microcomputer Tomography (Micro-CT) Analysis

Each sample was mounted with dental plaster in a plastic pipe to fabricate a repositioning index for micro-CT scanning. The sample position provided the direction of the carious lesion toward the pulp that was perpendicular to the scan axis (Figure 1(a)). To indicate the region of interest (ROI) with an approximate area of 0.03 mm^2^, a clear acrylic sheet with a hole size of Ø 0.1 mm was placed over the plastic pipe. A resin composite block (3 × 3 × 1 mm^3^) was attached to the exterior surface of the sample in order to locate the ROI obtained from micro-CT analysis when the samples were sectioned for field emission scanning electron microscope and energy-dispersive X-ray spectroscopy (FESEM-EDS) analysis (Figure 1(b)).

Before and after SDF application, each sample was scanned using a high-resolution micro-CT scanner (*µ*CT35; Scanco Medical AG, Bassersdorf, Switzerland) with an 18.5 *µ*m voxel size at 70 kVp voltage and 114 *µ*A current to measure (i) the mineral density profile (mgHA/cm^3^) at each 18.5 *µ*m depth interval from the lesion surface to the pulp, and (ii) the mineral density profile of the self-controlled sound dentin on the opposite side to the lesion. The scanning was performed under a relative humidity of 100%.

The estimated lesion depth was defined as the distance from the lesion surface to the point at which the baseline mineral density (MD_Before_) was up to 90% of the mineral density of the self-controlled sound dentin (MD_Sound_) at the same distance from the pulp surface. The estimated silver penetration depth was defined as the distance from the lesion surface to the point at which the percentage change in mineral density between MD_Before_ and MD_After_ was more than ten. The percentage change was calculated using the following formula:

(1) $$\begin{array}{c} \%\;{\text{Change}}_{\text{SDF application}}=\left(\frac{{\text{MD}}_{\text{After}}-{\text{MD}}_{\text{Before}}}{{\text{MD}}_{\text{Before}}}\right)*100. \end{array}$$

### 2.5. FESEM Observations and EDS Point Analysis

To prepare FESEM specimens, the root portion of each sample was removed. Then, each crown was fixed according to the previous studies' protocol, immersed in 2.5% glutaraldehyde for 2 h at 4°C followed by a 0.1% osmium tetroxide solution for 2 h at 4°C, and then dehydrated in an ascending ethanol series [19, 20]. Subsequently, each crown was embedded in clear resin and sectioned through a resin composite block using a low-speed diamond saw (Isomet1000; Buehler Ltd., Lake Bluff, IL, USA). The surfaces of the sectioned slices were polished with 600, 800, 1000, and 1200 SiC paper. Three specimens per group (one specimen per caries classification) were randomly selected to be imaged using FE-SEM (Apreo; FEI Co., Eindhoven, Netherland) without coating in a backscattered electron mode with 20 kV at 250x and 3500x magnifications (*n* = 6). The elemental spectrum of precipitating particles was identified using EDS-point analysis.

### 2.6. Statistical Analysis

The Mann–Whitney *U* test was used to compare the silver penetration depth/lesion depth (PD/LD) ratios between the two groups. All analyses were conducted using the STATA version 13.1 (StataCorp, College Station, Texas) with the significance level set at 0.05.

## 3. Results

### 3.1. Micro-CT Analysis

**Figure 2:**
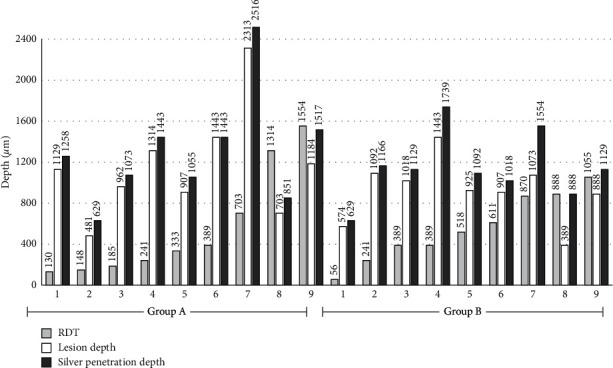
The estimated remaining dentin thickness (RDT), lesion depth, and silver penetration depth for each sample determined from micro-CT analysis (*N* = 18).

MD_before_ was subcategorized into demineralized dentin and sound dentin. The median mineral density values of demineralized dentin ranged from 3.91 to 941.59 mgHA/cm^3^, ascending from the outer toward the inner carious lesion zone. However, the median mineral density of sound dentin varied between 314.97 and 1220.38 mgHA/cm^3^, descending from the outer dentin toward the pulp. The estimated lesion depths and remaining dentin thickness (RDT) are shown in Figure 2.

After SDF application, there was an increase of 100–500 mgHA/cm^3^ mineral density (MD_after_- MD_before_) for each slice in both groups. However, it was observed that the continuation of increasing mineral density of demineralized dentin was limited to the dentin underneath the carious lesion where its MD_before_ was greater than MD_sound_. In all samples except sample no.6 in group A, the silver penetration depth was greater than the lesion depth. The micro-CT analysis revealed a favorable correlation between the silver penetration depth and the lesion depth (Figure 2).

The PD/LD ratios of group B (1.07–2.29) were marginally greater than those of group A (1.00–1.31). However, a statistically significant difference (*p*value = 0.5078) was not observed. When stratified by RDT, the PD/LD ratios of group B (1.12–2.29) were still greater than those of group A (1.09–1.19) when RDT >500 *µ*m. In contrast, there was no difference between groups when RDT was ≤500 *µ*m (Table 1).

Moreover, this study observed that silver particles precipitated in the pulp among samples with RDT <200 *µ*m, except for sample no. 3 in group A, which presented translucent dentin underneath the carious lesion.

### 3.2. FESEM Observations and EDS Point Analysis

**Figure 3:**
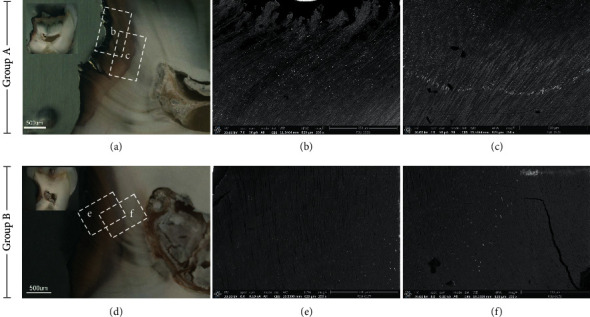
Cross-sectional stereomicroscope images of carious lesions treated with SDF and corresponding SEM micrographs at 250x magnification in group A (a–c) and B (d–f).

**Figure 4:**
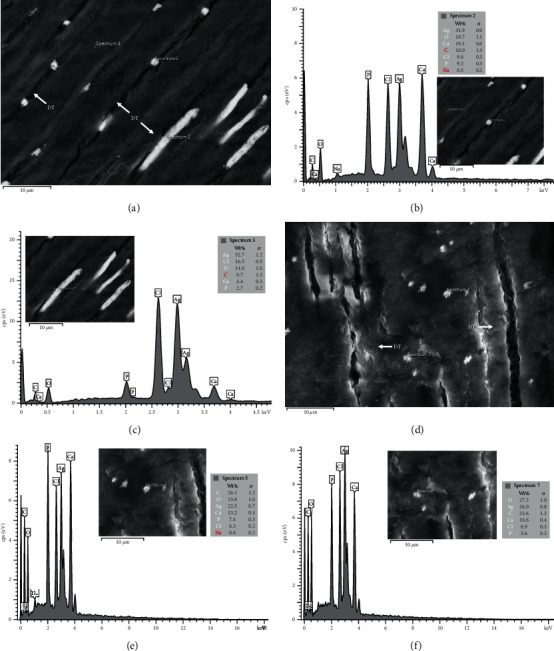
SEM micrographs at 3500x magnification and corresponding EDS point analysis at bright particles precipitated within tubular (a–c) and intertubular dentin (d–f).

The cross-sectional FESEM micrographs in both groups showed the bright particle distribution area associated with the extension of carious lesions as well as the increasing mineral density area assessed by a micro-CT (Figures 3(a)–3(f)). The results revealed that SDF could penetrate through necrotic tissue (Figure 3(b)) and precipitate within the underlying demineralized dentin. The precipitation of silver particles occurred within tubular and intertubular dentin (Figures 4(a) and 4(d)). The corresponding EDS point analysis on the bright particles indicated silver and other element spectrums such as chloride, phosphorus, and oxygen (Figures 4(b) and 4(c), 4(e), and 4(f)). The analyses suggested that the increased mineral density was a result of the silver compounds penetrating and precipitating.

## 4. Discussion

SDF is a potential adjunctive agent for deep caries management, but leaving carious tissue in the pulpo-proximal areas to avoid pulp exposure [1, 2] may affect the SDF penetration into the lesions. Silver penetration is critical for safety and effectiveness in this aspect. The evidence on this issue has been reported only in primary teeth [16]. Therefore, this study assessed the penetration of silver into carious lesions in permanent teeth treated with 38% SDF when necrotic dentin or soft dentin remained in pulpo-proximal areas.

The null hypothesis could not be rejected based on the findings of this investigation. This study demonstrated that leaving necrotic dentin in pulpo-proximal areas had no effect on silver penetration even when SDF was applied to a carious lesion with RDT >500 *µ*m. Silver penetrated through necrotic infected dentin and precipitated throughout the entire thickness of carious lesions. Additionally, it entered through the underlying sound dentin in a majority of lesions. However, the penetration depth of silver into sound dentin beneath the carious lesions was impacted by the amount and features of the remaining dentin.

To our knowledge, this is the first *in vitro* study to evaluate the penetration depth of silver in deep carious lesions in human permanent teeth. A dentin-pulp complex defensive response results in intratubular dentinogenesis or precipitation of dissolved minerals affecting dentin permeability [21]. The primary strength of this study is that the use of naturally occurring deep carious lesions with complex microstructures provides more clinical relevance. A micro-CT is a nondestructive testing method; therefore, outcome measurements before and after the SDF application are feasible. Another strength of this work is the estimation of lesion depth and silver penetration depth by comparing mineral density profiles within each sample. As a result, variations between samples could be controlled. In contrast, a previous study used a mean mineral density profile to calculate the lesion depth and mineral gain of demineralized enamel lesions. The enamel lesion depth was determined by comparing the baseline mineral density with the referenced maximum mineral density [22]. In this study, a cut-off point of 10% was used to differentiate the mineral density between sound and demineralized dentin or between silver precipitation and nonprecipitation. This value was derived from the measurement errors of repeated measures. However, due to the limitations of *in vitro* study, the increase in mineral density after the SDF application in this experiment is mainly a result of silver particle precipitation which may not resemble the actual remineralization process.

Microscopic structures in dentin consist of numerous water-filled dentinal tubules that provide diffusion channels to the dental pulp [21]. Dentin near the pulp is more permeable due to an increase in tubular density and diameter [21, 23]. The number of tubules increased from 15,000 to 20,000 tubules/mm^2^ at the DEJ to 45,000–60,000 tubules/mm^2^ at the dentin close to the pulp while the tubular size increased to 2-3 *µ*m in diameter at the pulp surface area [24, 25]. This study involved deep carious lesions that extended to the inner third of the dentin. Consequently, the penetration of water-soluble SDF through these lesions occurred easily due to the high permeability and moisture content in deep carious lesions.

Intervention in this study was based on strategies of carious tissue removal in deep caries as recommended by the International Caries Consensus Collaboration [1, 2]. Group A simulated a condition when pulpally soft caries removal was not feasible. Necrotic tissue consists of decomposed dentin contaminated with a great number of bacteria [26, 27]. Leaving necrotic dentin pulpally might reduce penetration of SDF to the lesions, similar to the effect of the smear layer during self-etch resin adhesive applications. The amount of silver ions bound to the necrotic tissue may lead to a decrease in free silver ion concentration. This might reduce the efficacy of SDF. This study showed that the removal of necrotic infected dentin (group B) tends to enhance silver penetration when RDT >500 *µ*m. However, no effect was found when SDF was applied to lesions extending to dentin near the pulp (RDT ≤500 *µ*m), which could be explained by the high permeability of dentin facilitating SDF penetration. The finding suggests that RDT is an important determinant for silver penetration. As a result, when SDF is applied to deep carious lesions, the amount of RDT should be considered.

In our study, when RDT <200 *µ*m, silver was able to penetrate through deep dentin and reach the pulp. A prior *in vivo* study in healthy pulp demonstrated that an RDT of ≥500 *µ*m is necessary to avoid pulp injury [28]. SDF was found to be cytotoxic to rat pulpal-like cells and inhibited alkaline phosphatase activity in another recent study [29]. In contrast, an *in vivo* study using SDF as an indirect pulp treatment material in artificial deep cavities (RDT≈ 250–500 *µ*m) showed that neither inflammatory change nor change in pulp histology occurred. Additionally, it was illustrated that SDF was more successful than calcium hydroxide at inducing tertiary dentin formation at 6 weeks after treatment when performed in healthy pulp conditions [30]. Silver in its different chemical forms may have varying degrees of cytotoxicity on pulpal cells. Silver chloride (AgCl) has lower cytotoxicity than SDF or silver ion according to previous research [31, 32]. Therefore, further studies regarding the pulpal response to SDF application in teeth with compromised dental pulp tissue conditions should be pursued.

In addition, our study observed that silver did not reach the pulp in one sample with an RDT of 185 *µ*m, while silver particles were found within the pulp in other teeth with an RDT of <200 *µ*m. This may be due to the presentation of translucent dentin at the base of the carious lesion blocking the silver penetration. In fact, the characteristic features of carious dentin vary dramatically from lesion to lesion. The inner carious dentin may consist of irregularities, less tubular structure, and partial or complete obstruction with mineral crystal deposits. Most samples in this study showed that the silver penetration depth was limited by sclerotic dentin, as indicated by a higher MD_before_ as compared to MD_Sound_ in micro-CT measurement. The sclerotic dentin was the result of the dentin-pulp response to noxious stimuli, producing a natural barrier to protect the pulp. The quality of dentin sclerosis formation depends on the caries progression rate and the activity of the pulp response [33]. Slow progressing lesions may reduce the chance of silver reaching the pulp. Therefore, the aspect of the caries progression rate should be considered thoroughly before the application of SDF in deep carious lesions in permanent teeth.

Silver precipitation in tubular and intertubular dentin might be due to the histological characteristics of the carious dentin. Loss of mineral content from peritubular and intertubular dentin provides transdentinal and intradentinal movement of SDF through the lesions. Once penetrated into demineralized dentin, SDF turns that area into an alkaline environment, facilitating the formation of covalent bonds between the phosphate groups and collagenous proteins. This interaction provides the apatite nucleation on the collagen, leading to remineralization of demineralized dentin [34, 35]. The SDF penetration throughout the entire thickness of carious lesions allows the antimicrobial effect, which is mainly derived from the silver element [9, 12, 36], to occur thoroughly. The exposure of reversible denatured collagen results in the precipitation of silver particles within demineralized carious dentin due to the presence of functional groups with a high affinity for silver ions, such as sulfur and nitrogen groups [19, 37]. Silver phosphate (Ag_3_PO_4_), silver chloride (AgCl), and silver protein complexes have been identified as the reaction products of SDF with tooth tissue in previous research [38, 39]. Ag_3_PO_4_ was a major reaction product which could turn into other compounds and release phosphate ions to initiate apatite formation.

(2) $$\begin{array}{c} {\text{Ca}}_{10}{\left({\text{PO}}_{4}\right)}_{6}{\left(\text{OH}\right)}_{2}+\text{Ag}{\left({\text{NH}}_{3}\right)}_{2}⟶{\text{CaF}}_{2}+{\text{Ag}}_{3}{\text{PO}}_{4}+{\text{NH}}_{4}\text{OH} \end{array}$$

Calcium fluoride (CaF_2_) is also a result of the chemical interaction between SDF and hydroxyapatite. CaF_2_ is well-known as an effective mineral reservoir for regulating the caries process and promoting dentin remineralization [40]. The increase in mineral density seen in this study following SDF treatment could be a result of not only silver compounds but also other compounds such as CaF_2_ precipitation. Due to the low atomic number and concentration of fluoride ions, our study was unable to identify fluoride penetration and precipitation in dentin carious lesions following SDF administration. Previous studies also failed to detect CaF_2_ precipitation in carious lesions following the application of SDF. This could be due to the low stability of CaF_2_, leading to its removal when rinsed with water [38, 39].

Compared with two existing studies on natural carious lesions in primary teeth, the penetration depth of silver detected in this study using permanent teeth (629–2516 *µ*m) was in agreement with the reported depth of 17-2490 *µ*m in primary teeth [16]. However, the results in this study were greater than those reported in another study with penetration depth limited to only 25–200 *µ*m [41]. Both studies were performed with no carious tissue removal. In our study, the results of using SDF with no carious tissue removal conditions indicated that leaving a large amount of necrotic infected dentin pulpally does not interfere with silver penetration into deeper parts of the lesions. This condition could be applied in the nonfunctional area which is hard to access or is in the first stage of stepwise excavation. A previous clinical study showed that there was no difference in the caries-arresting rate after 38% SDF application between no carious tissue removal and selective removal of soft dentin groups. However, that study was performed on carious lesions in primary upper incisor teeth with open cavities for self-cleansing nonrestorative treatment. The carious lesions were applied with SDF at 12-month intervals and followed for 30 months [42]. These factors might affect the caries-arresting rate. Thus, the different results among studies may be related to differences in carious tissue removal, measurement methods, baseline lesion depth, and pulp pressure presentation.

This study has some limitations. The absence of an intrapulpal pressure simulation might cause some differences between clinical and laboratory conditions. More research with a larger sample size is needed to validate silver penetration in deep carious lesions when (i) dentinal fluid flow is present as well as when (ii) RDT and (iii) caries activity are present. In addition, pulpal response following SDF administration should be investigated in teeth with damaged dental pulp tissue conditions.

## 5. Conclusion

Silver penetration is highly dependent on the amount and characteristics of remaining dentin beneath the carious lesion in relation to dentin permeability. Leaving necrotic dentin in pulpo-proximal areas does not interfere with silver penetration into the lesions. SDF solution could be applied with selective caries removal even when a large amount of carious tissue remained.

## Figures and Tables

**Table 1:** The minimum (Min), median, and maximum (Max) values of each variable in groups A and B.

	Group A (*n* = 9)			Group B (*n* = 9)		
	Min	Median	Max	Min	Median	Max
LD (*µ*m)	481.0	1128.5	2312.5	388.5	925.0	1443.0
PD (*µ*m)	629.0	1258.0	2516.0	629.0	1128.5	1739.0
RDT (*µ*m)	129.5	333.0	1554.0	55.5	1054.0	518.0
PD/LD ratio						
Overall	1.00	1.12	1.31	1.07	1.18	2.29
RDT >500 *µ*m	1.09	1.21	1.28	1.12	1.27	2.29
RDT ≤500 *µ*m	1.00	1.12	1.31	1.07	1.10	1.21

LD = lesion depth, PD = penetration depth, RDT = remaining dentin thickness, group A: no removal of necrotic dentin, group B: selective removal to soft dentin.

## Data Availability

The data used to support the findings of this study are available from the corresponding author upon request.

## Supplementary Materials

Supplementary Materials Supplementary Table 1: the demographic data of each sample (*N* = 18).
