# Supplementary material for: Penetration of Silver Diamine Fluoride in Deep Carious Lesions of Human Permanent Teeth: An In Vitro Study
Source: Int J Dent. 2021 Dec 22;2021:3059129. doi: 10.1155/2021/3059129 (PMC8716243; doi:10.1155/2021/3059129)
Supplement: Supplementary Materials — Supplementary Table 1: the demographic data of each sample (N = 18). [file 3059129.f1.docx]

**Supplementary table 1.** The demographic data of each sample (N=18).

| **Groups** | **Sample no.** | **Aged** | **Tooth type** | | **Caries classification** | | |
| --- | --- | --- | --- | --- | --- | --- | --- |
|  |  |  | **Premolar** | **Molar** | **I** | **II** | **V** |
| **A** | 1A | 30 | / |  |  | / |  |
|  | 2A | 32 |  | / |  | / |  |
|  | 3A | 30 |  | / |  | / |  |
|  | 4A | 24 | / |  |  | / |  |
|  | 5A | 52 |  | / |  | / |  |
|  | 6A | 39 |  | / |  |  | / |
|  | 7A | 31 |  | / |  |  | / |
|  | 8A | 31 |  | / | / |  |  |
|  | 9A | 30 |  | / |  |  | / |
| **B** | 1B | 60 |  | / |  | / |  |
|  | 2B | 35 |  | / |  | / |  |
|  | 3B | 24 | / |  |  | / |  |
|  | 4B | 25 |  | / | / |  |  |
|  | 5B | 35 | / |  |  | / |  |
|  | 6B | 37 |  | / |  |  | / |
|  | 7B | 31 | / |  |  | / |  |
|  | 8B | 45 |  | / |  | / |  |
|  | 9B | 32 |  | / |  |  | / |
